# Cost-effectiveness of implementing HIV and HIV/syphilis dual testing among key populations in Viet Nam: a modelling analysis

**DOI:** 10.1136/bmjopen-2021-056887

**Published:** 2022-08-11

**Authors:** David Coomes, Dylan Green, Ruanne Barnabas, Monisha Sharma, Magdalena Barr-DiChiara, Muhammad S Jamil, R Baggaley, Morkor Newman Owiredu, Virginia Macdonald, Van Thi Thuy Nguyen, Son Hai Vo, Melanie Taylor, Teodora Wi, Cheryl Johnson, Alison L Drake

**Affiliations:** 1 Department of Epidemiology, University of Washington, Seattle, Washington, USA; 2 Department of Global Health, University of Washington, Seattle, Washington, USA; 3 Department of Medicine, University of Washington, Seattle, Washington, USA; 4 Department of Global HIV, Hepatitis and STI Programmes, World Health Organization, Geneva, Switzerland; 5 Viet Nam Country Office, World Health Organization, Hanoi, Viet Nam; 6 Viet Nam Authority for HIV/AIDS Prevention and Control, Government of Viet Nam Ministry of Health, Hanoi, Viet Nam; 7 Division of STD Prevention, Centers for Disease Control and Prevention, Atlanta, Georgia, USA; 8 Clinical Research Department, London School of Hygiene & Tropical Medicine, London, UK

**Keywords:** HEALTH ECONOMICS, HIV & AIDS, PUBLIC HEALTH

## Abstract

**Abstract:**

**Objectives:**

Key populations, including sex workers, men who have sex with men, and people who inject drugs, have a high risk of HIV and sexually transmitted infections. We assessed the health and economic impacts of different HIV and syphilis testing strategies among three key populations in Viet Nam using a dual HIV/syphilis rapid diagnostic test (RDT).

**Setting:**

We used the spectrum AIDS impact model to simulate the HIV epidemic in Viet Nam and evaluated five testing scenarios among key populations. We used a 15-year time horizon and a provider perspective for costs.

**Participants:**

We simulate the entire population of Viet Nam in the model.

**Interventions:**

We modelled five testing scenarios among key populations: (1) annual testing with an HIV RDT, (2) annual testing with a dual RDT, (3) biannual testing using dual RDT and HIV RDT, (4) biannual testing using HIV RDT and (5) biannual testing using dual RDT.

**Primary and secondary outcome measures:**

The primary outcome is incremental cost-effectiveness ratios. Secondary outcomes include HIV and syphilis cases.

**Results:**

Annual testing using a dual HIV/syphilis RDT was cost-effective (US$10 per disability-adjusted life year (DALY)) and averted 3206 HIV cases and treated 27 727 syphilis cases compared with baseline over 15 years. Biannual testing using one dual test and one HIV RDT (US$1166 per DALY), or two dual tests (US$5672 per DALY) both averted an additional 875 HIV cases, although only the former scenario was cost-effective. Annual or biannual HIV testing using HIV RDTs and separate syphilis tests were more costly and less effective than using one or two dual RDTs.

**Conclusions:**

Annual HIV and syphilis testing using dual RDT among key populations is cost-effective in Vietnam and similar settings to reach global reduction goals for HIV and syphilis.

**Strengths and limitations of this study**

- Our model parameters are informed by empiric data including demographic, behavioural and biological data from government sources, surveys, surveillance, publicly available reports, databases and peer-reviewed literature.
- We assess the impact of five testing scale up scenarios using both HIV rapid diagnostic test (RDT) and dual HIV/syphilis RDT and conduct sensitivity analyses to evaluate uncertainty in model results.
- Due to limited data, we make assumptions regarding the timing and uptake of HIV and syphilis testing among key populations that may be inaccurate.
- Our model conservatively assumes that increased syphilis testing and treatment will not impact syphilis prevalence, which is currently unknown.

## Introduction

 Key populations, including people who inject drugs (PWID), men who have sex with men (MSM), sex workers (SW) and transgender populations, are at higher risk of acquiring both HIV and syphilis. HIV incidence is significantly higher among key populations compared with the general population in all geographical regions; however, differences vary substantially by region and by key population.^1^ While key populations and their sexual partners represent approximately 25% of new HIV cases in sub-Saharan Africa, they represent 80% of new HIV cases in the rest of the world.^2^ Recent data suggests that syphilis incidence, while generally remaining stable in low-income and middle-income countries (LMICs), is increasing among key populations, particularly MSM.^1 3 4^ WHO HIV testing guidelines recommend HIV retesting at least annually for key populations and more frequent testing (3–6 months) for those with high ongoing risk.^5^ WHO guidelines for syphilis screening depend on population and setting. Laboratory-based syphilis testing remains common, however rapid diagnostic tests (RDTs) for syphilis are increasingly available and may be used to improve access to testing and treatment, including among key populations who are disproportionately affected by both HIV and syphilis.^6^ With the introduction of prequalified dual HIV/syphilis RDTs, and the recent WHO recommendation to offer dual HIV/syphilis testing in antenatal care (ANC) settings,^7^ it is important to evaluate how further integration and expansion of dual HIV/syphilis testing could benefit key populations.

Since 2015, WHO has recommended immediate initiation of antiretroviral therapy (ART) for all people living with HIV (PLWH)^8^ and the United Nations 95-95-95 targets aim to diagnose 95% of PLWH, provide 95% of PLWH who know their status with ART, and ensure 95% PLWH on ART are virally suppressed.^9^ Despite progress towards these goals—in 2019 81% of PLWH knew their HIV status and 67% were on ART—this progress is uneven; only two-thirds of key populations are aware of their HIV status.^2^ While key populations lag behind the general population in all phases of testing, linkage to treatment, and viral suppression, the largest gap exists in testing.^10^ WHO has also developed a global strategy on sexually transmitted infections (STIs) which aims for a 90% reduction in syphilis incidence by 2030, and 70% of key populations to have access to STI and HIV services, including prevention, testing, and treatment.^11^ Increased syphilis testing and treatment may reduce syphilis burden among key and general populations, as well as HIV incidence since early symptomatic syphilis increases risks of HIV acquisition and transmission.^3^ Currently, WHO recommends syphilis testing for pregnant women and key populations, however, the optimal frequency of syphilis testing is unknown and recommendations on syphilis testing for other populations are not available.

In Viet Nam, the national HIV prevalence is <1% in the general population, and significantly higher in key populations, with prevalence ranging between 3% and 13% among PWID, MSM and female SW (FSW). Similarly, syphilis prevalence among MSM (6.7%) and FSW (2.1%) are also higher than that of the general population (0.3%).^12^ With budgetary constraints in HIV/STI programmes and the health sector, identifying cost-effective strategies for targeted HIV and syphilis testing among key groups in Viet Nam is crucial to inform policymakers seeking to optimise resource allocation to maximise population health. We modelled the health impacts and costs associated with varying frequencies of HIV and syphilis testing for key populations, using test scenarios that include a dual HIV/syphilis RDT.

## Methods

### Model

We used the AIDS Impact Model within the Spectrum software package (V.5.76) to simulate the HIV epidemic in Viet Nam from 2020 to 2035. The model estimates annual HIV incidence, AIDS mortality and disability. We simulated the impact of increasing HIV testing frequency among key populations using the Goals model within Spectrum, as previously described.^13^ Briefly, Spectrum is a deterministic, compartmental mathematical model of HIV transmission stratified by sex and age. Transmission is simulated through male-female and male-male sex acts, needle sharing for injection, and maternal-to-child transmission with specific transmission probabilities for each route. One can further specify parameters for low- and medium-risk groups, as well as high-risk categories including FSW, MSM, and PWID. Low-risk heterosexuals are those in stable couples while medium-risk heterosexuals are those that engage in casual sex but are not in a high-risk group (high risk groups: MSM, FSW, PWID). Each of these high-risk categories is nested within their parent categories and interact with one another. For example, among MSM, a proportion is assumed to also have female sexual partners. These high-risk categories can be parameterised to have differential rates of partnership and uptake of interventions. The model was parameterised with demographic, behavioural and biological data from government sources, surveys, surveillance, publicly available reports, databases and peer-reviewed literature.

To estimate syphilis burden, we used key population size estimates from the Goals model and population-specific estimates of prevalence^12^; we estimate the number of persons in key populations testing positive and treated for syphilis infection under each scenario. This model assumes that syphilis testing and treatment does not impact syphilis prevalence, although increased screening could potentially result in reduced, unchanged or increased syphilis prevalence depending on coverage.^14 15^ For both HIV and syphilis, disability-adjusted life years (DALYs) are calculated for each scenario. Model key parameters are shown in table 1.

**Table 1:** Model parameters for spectrum input and cost-effectiveness analysis of HIV and syphilis testing scale up among key populations in Viet Nam

Model parameter	Value
HIV Prevalence	
MSM (incl. TGW)*	10.8%
PWID**	12.7%
FSW*	3.6%
Syphilis prevalence^12^	
MSM (incl. TGW)	6.7%
PWID	0.3%
FSW	2.1%
Baseline syphilis test acceptance	
MSM (incl. TGW)^16 49 50^	27%
PWID^17^	16%
FSW^18 51^	35%
Syphilis DALYs averted^52^	
DALYs averted per syphilis case treated	0.04
ART	
2019 coverage††	70%
Annual scale-up†	4.8%
Transmission reduction efficacy‡	70%
Mortality reduction efficacy‡	80%
Other prevention	
Condom use†	50%
Condom efficacy‡	80%
PrEP coverage (MSM incl. TGW)†	5%
PrEP efficacy‡	90%
PrEP adherence‡	80%
Costs†*	
HIV lay test§	$4.50
Syphilis RPR§	$6.28
Syphilis TPHA§	$10.26
HIV/syphilis dual test§	$6.50
ART¶	$285
Syphilis treatment	$6.50
Time horizon	2020–2035
Discount rate	3%

* 2018 Viet Nam HIV Sentinel Surveillance.

† Assumed.

‡ Spectrum model prior.

§ Testing costs include labour, incentives, travel costs and test kits. Primary cost driver between tests is the cost of the test kit.

¶ ART cost includes labour, laboratory monitoring costs, antiretroviral drugs (ARVs) and other recurring costs.

** 2019 Viet Nam HIV Sentinel Surveillance.

†† Based on information from in-country source.

ART, antiretroviral therapy; DALY, disability-adjusted life-year; FSW, female sex workers; MSM, men who have sex with men; PrEP, pre-exposure prophylaxis; PWID, people who inject drugs; RPR, rapid plasma reagin; TGW, transgender women; TPHA, treponema pallidum haemagglutination assay.

### Settings and populations

We modelled three key populations: MSM, PWID and FSW (and their clients) within the HIV epidemic in Viet Nam, using national level HIV prevalence and syphilis prevalence estimates for each key population (table 1).

### Scenarios

Our baseline scenario estimates annual HIV testing based on current WHO recommendations and estimated HIV testing rates among key populations,^5^ and syphilis testing based on observed uptake. In the baseline scenario, we assume 50% of individuals in key populations test annually for HIV, and syphilis screening with a non-treponemal test (rapid plasma reagin, RPR) occurs at rates specific to each key population (table 2).^16^,^18^ Individuals who test positive using RPR are given a confirmatory treponemal (treponema pallidum haemagglutination, TPHA) test. We modelled HIV RDTs as WHO recommend, limiting the use of lab-based testing such as western blot, especially in hard-to-reach populations, to increase access and limit the loss to follow-up.^19^ We considered alternative scenarios with varying testing frequency and test type (separate HIV and syphilis RPR, or a combined dual syphilis/HIV RDT) among key populations from 2020 to 2035. Scenarios modelled include: (1) annual HIV testing with RDT and baseline syphilis RPR testing, (2) annual testing with dual RDT, (3) biannual testing (two times per year), first with dual RDT and then with HIV RDT, (4) biannual HIV testing with RDT and baseline syphilis RPR testing and (5) biannual testing with dual RDT (table 2). We assumed 75% test acceptance for the first test in all scenarios except baseline, and 90% of those who accepted the first test would accept the second test in all scenarios that include biannual testing. All individuals who test positive for syphilis using the dual test are then tested using RPR and TPHA per current Viet Nam country guidelines.

**Table 2:** HIV/syphilis testing scenarios among key populations in Viet Nam

Scenario	Proportion of key population receiving HIV or syphilis testing per year			
	1 HIV test	2 HIV tests	1 syphilis test	2 syphilis tests
Baseline	50%	–	35% (FSW), 27% (MSM), 16% (PWID)	–
1. One HIV RDT	75%	–	35% (FSW), 27% (MSM), 16% (PWID)	–
2. One dual HIV/syphilis RDT	75%		75%	–
3. One HIV RDT and one dual HIV/syphilis RDT	75%	68%	75%	–
4. Two HIV RDTs	75%	68%	35% (FSW), 27% (MSM), 16% (PWID)	–
5. Two dual HIV/syphilis RDT	75%	68%	75%	68%

The table cells show the proportion of key populations in Viet Nam that receive each test per year. If not specified, the proportion refers to all key populations.

FSW, female sex workers; MSM, men who have sex with men; PWID, people who inject drugs; RDT, rapid diagnostic test.

We modelled increases in testing coverage by adjusting the percent of people living with HIV (PLWH) on ART; in scenarios with increased testing there is a higher probability that an individual in the model will initiate ART throughout the year. The baseline scenario assumes 95% ART coverage among PLWH by 2028 (4.8% increase per year) based on recent ART scale-up in Viet Nam; test coverage increases by 6.0% per year with annual HIV testing (HIV RDT or dual RDT), and by 7.2% per year with biannual testing. Maximum test coverage is 95% for each model. All models assume ART coverage of 66% of men and 72% of women living with HIV in 2020 based on estimates from the Viet Nam HIV-AIDS Technical Working Group. Modelled HIV incidence per year is shown in online supplemental figure S1. We assume universal treatment among those who test positive for syphilis, individuals treated cannot become renfected within the same year,^20^ and no changes to syphilis prevalence under test case scenarios.

**Figure 1:**
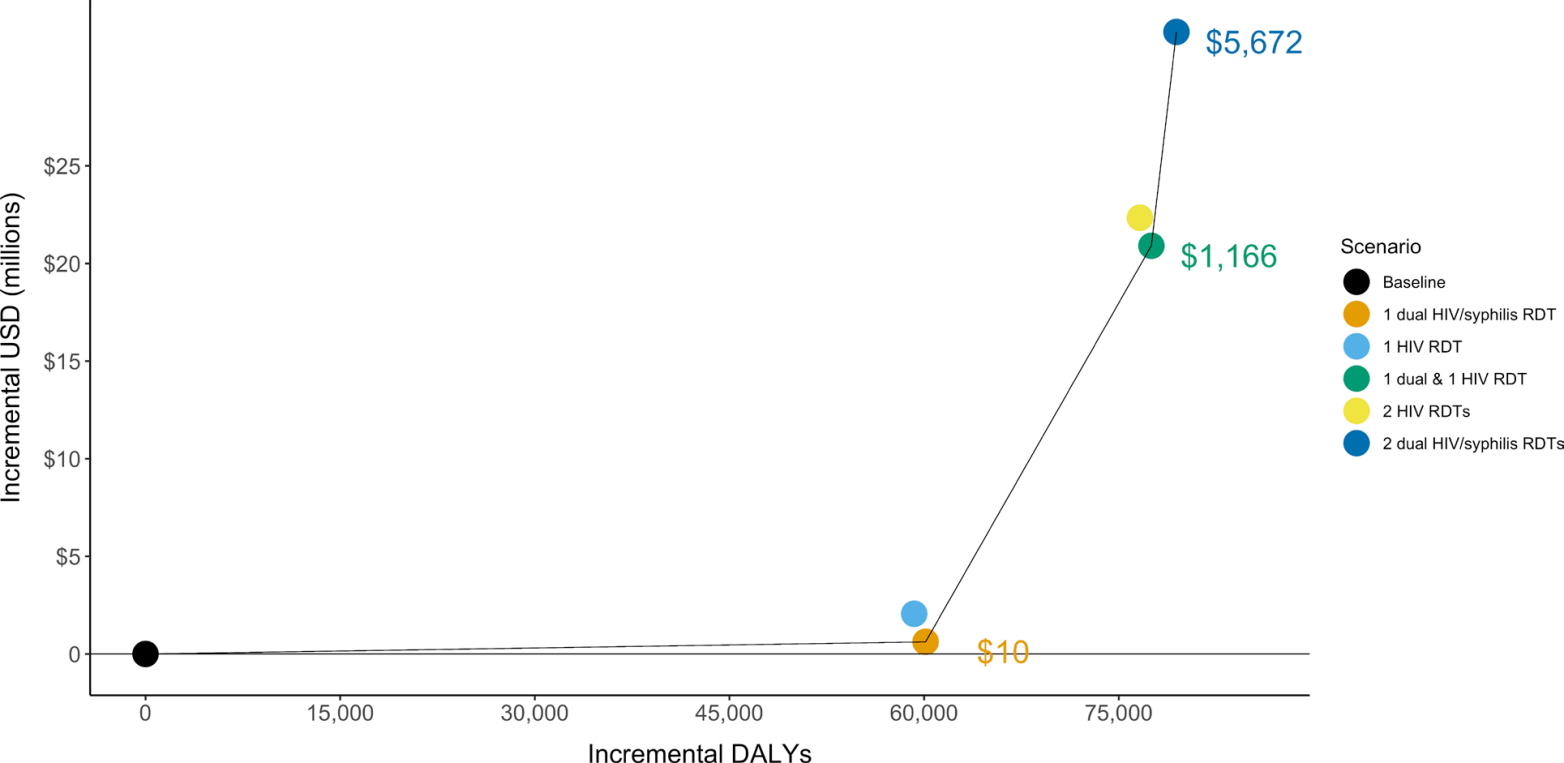
Efficiency frontier presenting the total disability-adjusted life-years (DALYs) and costs for five testing scenarios among key populations. The solid line indicates the scenarios that are not dominated by other scenarios. Dominated indicates that a scenario is either more costly and less effective or has a higher ICER than a scenario that is more effective. The ICERs for the non-dominated scenarios are shown. ICER, incremental cost-effectiveness ratio; RDT, rapid diagnostic test; .

### Costs

Testing cost inputs include cost per HIV RDT test, RPR, TPHA, and dual HIV/syphilis RDT. We used local data on the personnel, commodities and transport costs associated with lay testing and estimate costs (table 1). ART costs include personnel, commodities, clinical follow-up and laboratory monitoring. This analysis includes the costs of intervention delivery and treatment (Benzathine penicillin G and ART) but does not consider additional averted sequelae costs such as the treatment of opportunistic infections due to uncontrolled HIV. All costs are from the provider’s perspective and reported in 2019 US dollars.

### Cost-effectiveness

Health impact was measured in DALYs averted, HIV infections averted, syphilis infections treated and AIDS-related deaths averted over the 15-year time horizon. This time horizon was chosen because it reflects current HIV programme planning in Viet Nam. HIV outcomes are modelled for the entire population of Viet Nam while syphilis outcomes are specific to key populations. Costs and health benefits were discounted at 3% annually per standard health economic evaluations.^21^ Incremental costs were calculated as costs incurred and averted by the testing strategy. We uused WHO guidelines for cost-effectiveness threshold: less than gross domestic product per capita is considered cost-effective in Viet Nam (US$2715 in 2019).^22^

### Model calibration and sensitivity analyses

Models were calibrated to national HIV prevalence data for each key population. Monte Carlo sensitivity analyses were conducted to evaluate robustness of results to changes in: HIV and syphilis testing coverage, scenario programme uptake rate, HIV and syphilis testing cost, HIV and syphilis treatment cost, average years on ART and time horizon. Online supplemental table S1 shows the model parameters, ranges and distributions used in the sensitivity analysis.

### Patient and public involvement

It was not appropriate or possible to involve patients or the public in the design, conduct, reporting or dissemination plans of our research.

## Results

Increasing annual HIV test coverage from 50% (baseline) to 75% using an HIV RDT (scenario 1) or a dual RDT (scenario 2) is projected to avert 3,206 HIV infections and 660 AIDS-related deaths by 2035 in Viet Nam (table 3). Annual testing using dual RDT led to treatment of an additional 27 727 syphilis cases over 15 years compared with using HIV RDT, but the number of HIV infections averted was the same. HIV testing with either HIV or dual RDT biannually (scenarios 3, 4 and 5) was projected to avert an additional 875 HIV infections and 183 AIDS-related deaths by 2035 compared with annual testing. Testing using a dual HIV/syphilis RDT biannually among key populations is projected to lead to an additional 60 674 syphilis cases treated by 2035, compared with annual testing using a dual RDT.

**Table 3:** Estimated HIV and syphilis infections, and cost-effectiveness of increased HIV and dual HIV/syphilis testing among key populations in Viet Nam from 2020 to 2035

		Scenario					
		Baseline	1 dual test	1 HIV test	1 HIV and 1 dual	2 HIV tests	2 dual tests
HIV	New HIV infections	57 902	54 696	54 696	53 821	53 821	53 821
	AIDS deaths	13 877	13 217	13 217	13 034	13 034	13 034
	Total HIV DALYs	174 567 240	174 508 007	174 508 007	174 490 608	174 490 608	174 490 608
Syphilis	Total cases treated	88 953	116 680	88 953	116 680	88 953	177 354
	Total DALYs treated	2831	3713	2831	3713	2831	5644
Incremental cases averted	HIV infections averted	–	3206	0	875	0	0
	HIV DALYs averted	–	59 233	0	17 399	0	0
	Syphilis cases treated	–	27 727	−27,727	27 727	−27,727	88 401
	Syphilis DALYs averted	–	882	−882	882	−882	2813
Total DALYs averted (HIV and syphilis)		–	60 115	−882	18 281	−882	2813
Costs(USD)	Net costs	$31 036 672	$31 659 182	$33 094 783	$51 942 954	$53 378 555	$62 896 039
	HIV testing	$16 491 955	–	$24 683 204	$22 142 027	$46 825 230	–
	HIV treatment averted	–	-$6 133 138	-$6 133 138	-$7 991 393	-$7 991 393	-$7 991 393
	Syphilis testing	$14 084 698	$1 535 409	$14 084 698	$1 535 409	$14 084 698	$2 333 826
	Syphilis treatment	$460 019	$603 395	$460 019	$603 395	$460 019	$917 162
	Dual testing	–	$35 653 516	–	$35 653 516	–	$67 636 444
Total incremental costs		–	$622 510	$1 435 601	$18 848 171	$1 435 601	$9 517 484
ICERs (cost per DALY averted)		–	$10	*Dom*	$1166	*Dom*	$5672

Each scenario refers to the number of tests per year. The baseline scenario assumes that 50% of key populations are tested for HIV each year and syphilis testing rates are specific to each subpopulation (FSW, MSM and PWID). Scenarios including one test per year assume a 75% test acceptance rate, and those that include two tests per year assume a 75% test acceptance rate for the first test, and a 68.5% test acceptance rate for the second test. Incremental cases averted, total DALYs averted and ICERs compare each scenario to the previous one.

DALY, disability-adjusted life-years; FSW, female sex workers; ICERs, incremental cost-effectiveness ratios; MSM, men who have sex with men; PWID, people who inject drugs.

The most effective strategy was biannual testing among MSM, PWID, and FSW with the dual RDT, which was projected to avert 4,081 HIV infections (7% of total infections), 76,632 HIV DALYs (0.04% of total HIV DALYs) and treat 88 401 cases of syphilis by 2035 compared with the baseline scenario. The discounted cost of implementing this scenario over 30 years is US$62.9 million compared with US$31.0 million for the baseline scenario. The testing cost of implementing biannual testing using the dual RDT is approximately four times the cost of baseline testing with an HIV RDT (US$67.6 million vs US$16.5 million, respectively), but an estimated US$8.0 million in HIV treatment costs would be averted by biannual HIV testing and US$11.8 million in syphilis testing costs would be averted by using the dual RDT. The cost of biannual testing with an HIV RDT and continuing to test for syphilis with RPR is higher than biannual testing with one dual RDT and one HIV RDT (US$53.4 vs US$51.9 million USD, respectively), but using one dual test in biannual testing treats an estimated 28 000 more cases of syphilis over 15 years while averting the same number of HIV cases.

Annual testing with the dual RDT is cost-effective compared with the baseline scenario (US$10 per DALY averted) (figure 1). Annual HIV testing with HIV RDT is more expensive and averts fewer DALYs than with the dual RDT (strongly dominated). The next most efficient scenario is biannual testing using one dual RDT and one HIV RDT, which is cost-effective (US$1166 USD per DALY averted). Biannual testing with HIV RDT is less effective and more costly than biannual testing using one dual RDT and one HIV RDT, while biannual testing using the dual RDT provides additional health benefits but is not cost-effective (US$5672 USD per DALY averted). Despite slightly higher initial costs, the discounted cost of annual testing with a dual RDT becomes less than that of current testing within 2 years, due to decreased ART costs associated with HIV averted (online supplemental figure S2).

**Figure 2:**
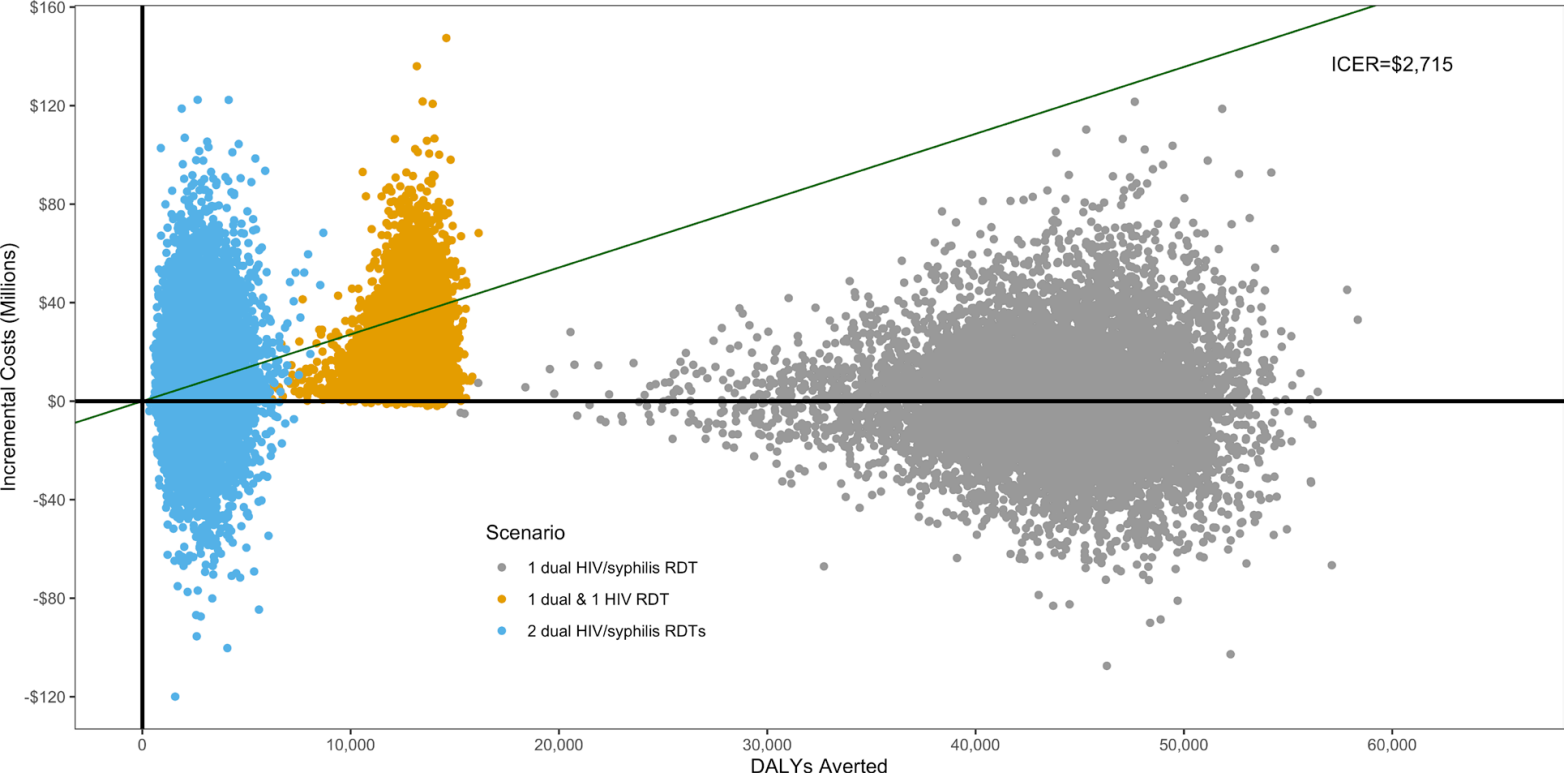
Sensitivity analysis of non-dominated scenarios using a Monte-Carlo simulation of the cost-effectiveness of HIV/syphilis dual testing among key populations in Viet Nam. Plot shows 10 000 iterations in which 17 key parameters were randomly adjusted. All points below the green line are cost-effective at US$2715 per DALY averted and those below the solid black line (y-intercept) are cost saving. Only non-dominated scenarios are shown in this figure; cost-effectiveness of 1 dual test is compared with baseline, 1 HIV test and 1 dual test is compared with 1 dual test, and 2 dual tests is compared with 1 HIV test and 1 dual test. DALYs, disability-adjusted life-years; ICER, incremental cost-effectiveness ratio; RDT, rapid diagnostic test.

Sensitivity analyses including all scenarios found that an annual dual RDT (scenario 2) is cost-saving in 52% of the simulations and either cost-saving or cost-effective (at US$2715 per DALY averted) in all simulations (online supplemental table S2). Biannual testing using one dual RDT and one HIV RDT was cost-effective in 86% of simulations, but cost saving in only 1% of simulations (figure 2). Biannual testing using two dual RDTs was cost-effective in 45% of simulations and cost saving in 31% of simulations as compared with biannual testing with one dual RDT and one HIV RDT. In univariate sensitivity analysis adjusting costs, our scenarios that involve one dual RDT (scenarios 2 and 3) remain cost-effective even after all costs (testing and treatment) are increased by 50% (US$16 and US$1705 USD per DALY averted, respectively).

## Discussion

In this modelling analysis, we found that implementing annual testing among key populations with the dual RDT at 75% coverage was cost-effective, averted more HIV infections and treated more syphilis cases compared with annual testing using HIV RDT at 50% coverage and current syphilis testing in Viet Nam. While biannual testing with one dual RDT and one HIV RDT was projected to be more costly, it would avert more HIV and syphilis related DALYs, and using dual RDT for both tests would avert additional DALYs attributed to syphilis, although this latter scenario was not found to be cost-effective. Increasing the frequency of HIV testing to one or two tests per year using only HIV RDTs (scenarios 1 and 4), while continuing to screen for syphilis using RPR, was not efficient compared with other strategies.

Implementing biannual testing substantially increases testing costs, but also prevents more HIV infections, therefore averting more HIV healthcare costs, including ART and hospitalisations. Increasing test frequency may be cost saving or cost-effective, although it incurs considerable costs in the near term while costs averted may not be observed for many years. Annual testing using a dual RDT can help offset some near-term costs as it is less expensive than using HIV RDT and syphilis RPR. Policy-makers must weigh the health impact and cost-effectiveness of different testing scenarios over time against current affordability; however, using the dual RDT will help integrate syphilis testing within existing HIV testing programmes, improving programme efficiencies.^23^

Implementation of dual RDT is occurring in some settings; preliminary reports indicate that 49 countries have adopted policies to use dual HIV/syphilis RDT in ANC, and 15% of reporting counties have policies to support their use in key populations, although the extent of implementation is unknown.^24^ The President’s Emergency Plan for AIDS Relief and the Global Fund both fund dual RDT in ANC,^25^ and there are multiple dual RDTs prequalified by the WHO.^26^ The use of dual RDT during ANC could be a model for improving HIV/STI integration, particularly among those at high risk for both HIV and syphilis, such as key populations, however, there are operational challenges associated with integrating HIV and STI programmes and delivering person-centred diagnosis, treatment and prevention services.^27^

Benefits of the dual test are its potential to cost-effectively reach more at-risk individuals at the point of care. Annual or biannual testing can enable earlier identification of HIV-positive individuals for faster ART initiation and prevention of onward transmission. Annual HIV testing for key populations is recommended by WHO, and more frequent testing (every 3–6 months) may be advised for those with individual risk factors, including those using pre-exposure prophylaxis (PrEP) and key populations presenting with STIs.^19^ Individuals presenting with syphilis symptoms should also test for HIV, and using the dual RDT is less costly as compared with a syphilis RPR and HIV RDT. As policy-makers scale up PrEP among key populations in Viet Nam, including at least one dual RDT in the testing algorithm may be more cost-effective than using HIV RDTs alone. In addition, using dual RDT tests can facilitate lay providers to offer both HIV and syphilis testing for their community.^28^

Our results were robust to sensitivity analyses, suggesting that testing annually or biannually using dual RDTs remains cost-effective if testing costs increase and HIV prevalence decreases. In scenarios involving dual RDT, the majority (>98%) of benefits, as measured in DALYs, come from averting HIV infections rather than treating syphilis due to the relatively large burden of disease from HIV compared with syphilis. However, since the cost of a dual RDT is only slightly higher than the cost of an HIV RDT, it is cheaper to use a dual RDT than separate HIV RDT and syphilis RPR tests in situations where both tests are recommended.

Increased HIV testing can reduce HIV-associated morbidity and mortality and transmission from PLWH through early detection and initiation of ART. While models suggest high ART coverage would result in substantial declines in HIV incidence,^29 30^ empiric data from countries with population-level viral suppression exceeding 73% (eg, Australia, eSwatini and Thailand) have observed less significant reductions in HIV incidence relative to predictions from mathematical models.^31^ Similarly, when high ART coverage was achieved in a series of cluster-randomised trials in sub-Saharan Africa, it resulted in decreased population-level HIV incidence; however, this decrease was insufficient to end HIV as a public health threat.^32^,^35^ These discrepancies may in part be attributed to delayed diagnosis and ART initiation following infection,^36 37^ and gaps in the 95-95-95 targets for some population groups, for example young men and key populations. Additional barriers may include poor coverage of evidence-based prevention interventions and persistent structural barriers, particularly for key populations. More frequent HIV testing strategies could increase earlier diagnosis and initiation on ART and focusing testing and linkage efforts on key populations could reduce the access and coverage disparities in these groups. However, more frequent testing will also increase programme costs, not only through additional commodity procurement but also for health systems, programme coordination, and outreach. Policy-makers may likely benefit from targeting limited testing resources towards high-risk groups such as key populations.

Dual RDTs may also increase syphilis testing frequency and coverage among key populations who are more likely to access HIV testing than testing for syphilis. Previous research has shown that coupling rapid syphilis testing in ANC may also increase HIV test coverage in LMICs, particularly in settings where HIV test coverage is low.^38^ This strategy may be similarly effective at increasing test coverage for both diseases among key populations, as well as augment current ANC testing by reaching women in key populations who present late or are missed by ANC services. While there is a lack of data on dual RDTs among key populations, models of dual RDT during ANC have been shown to be cost saving or cost-effective among both key populations and the general population of pregnant women.^7 39^ While dual RDTs are likely more effective in the context of ANC since testing can avert more adverse outcomes associated with congenital syphilis and mother-to-child HIV transmission, we find dual RDTs may also be cost-effective among non-pregnant key populations.

Our results are consistent with previously published models that show expanded testing and early access to ART for key populations in Viet Nam will cost-effectively reduce the country’s HIV burden.^40 41^ Additionally, models from both low-resource and high-resource countries suggest HIV testing every 3–6 months among key populations can be cost-effective in concentrated epidemics.^42 43^ However, HIV risk within key populations is not homogenous; further targeting of higher-risk groups within key populations may be needed to achieve efficient testing regimens. While we examine the impact of increased testing frequency among key populations as a whole, previous research has described the benefits of targeting high-risk groups within key populations.^44^ Individuals who engage in risky behaviours, such as those with more sexual partners, practicing unprotected sex or needle/syringe sharing may benefit from additional testing or linkage to HIV prevention such as PrEP and harm reduction interventions. Further research is needed on the optimal testing intervals for higher-risk groups of key populations.

Globally, approximately one-third of key populations are not aware of their HIV status. Programmes focusing on HIV testing and treatment among FSW and PWID in Viet Nam have shown success in reducing HIV prevalence in these groups; however, less than a third of MSM reported testing for HIV in 2015, likely contributing to increases in HIV prevalence among this group in the past decade.^45^ Annual syphilis testing among key populations in Viet Nam is similarly low, ranging from 16% among PWID to 36% among FSW.^16^,^18^ Due to high dual prevalence of HIV and syphilis among key populations, dual testing is a promising strategy to increase testing coverage and linkage to care.

Our analysis has several limitations. We did not include the cost of scaling-up and training providers in administering dual RDTs. However, RDT are easy to use and can be administered by a lay provider, and rapid results can minimise loss to follow-up. Overall, dual RDTs have been shown to have adequate performance in field settings in Viet Nam among key populations.^46^ Dual RDTs may also increase HIV test coverage as they can be easily conducted by community health workers outside of healthcare settings, and they may be more acceptable to some members of key populations who are concerned about stigma associated with testing.^47^ Dual RDTs may also expand syphilis testing uptake, as most syphilis cases in Viet Nam are currently diagnosed at provincial hospitals. Despite this, some additional training, supervision and support will be needed to scale-up dual RDT use among key populations.

We did not explicitly model HIV testing or diagnosis in this analysis as HIV testing uptake is not an adjustable model parameter in Spectrum. We instead modelled ART coverage, which required assumptions about the link between testing frequency and ART coverage. Since data on the impact of retesting on population HIV incidence is limited, we made conservative assumptions about the frequency of linkage to care and ART use following retesting. We assumed that HIV testing frequency would increase in Viet Nam among key populations in the baseline scenario but testing frequency would increase more quickly under the other scenarios. Because of this, we believe our estimates of the impact of increased testing frequency are conservative. Due to the lack of evidence on the impact of retesting on population HIV incidence, a model that explicitly includes testing rates as a parameter would also need to rely on assumptions concerning the relationship between testing behaviour and ART enrollment.

Some model assumptions regarding the timing of HIV and syphilis testing may be inaccurate. Timing of testing is an important component from both a technical analytical perspective and guideline development process. In truth, there are a nearly limitless number of permutations of frequency and spacing of retests. We chose even spacing as it is easily interpretable at all levels of research, policy, and service delivery. This maximal spacing between tests is expected to have the largest impact at the population level, assuming risk is evenly spread across the calendar year. We assume regular testing intervals for the entire population in each scenario, but it is possible—and entirely sensible—for people who had a risky sexual encounter or who are experiencing symptoms to seek more frequent retesting than biannually. We assumed in scenarios that included a dual RDT, additional syphilis screening tests would not be conducted. However, PLWH who know their status and present for syphilis screening do not need an HIV test.

We did not include the costs of outreach to achieve increased test coverage of key populations. Considerable expansions of first time testing among MSM in Viet Nam have recently been achieved through social media campaigns, perhaps providing a guide for cost-effectively increasing testing uptake among key populations.^45 48^ We also did not consider the burden that increased test coverage and frequency may have on the health system; however, as testing may be conducted effectively using lay providers, increased testing may not substantially impact the provision of other services.^47^ Although targeting key populations in lower prevalence regions may be more difficult and costly, these results are robust to increased costs and it will likely remain an effective use of resources. Research that focuses on province-specific estimates of cost and impact would likely find that focusing on high-burden areas is more cost-effective; however, health policy, financing, guideline development and implementation continue to be led nationally in Viet Nam. Therefore, national-level evidence is needed to direct decision making.

We assume that syphilis screening will not impact syphilis prevalence rates. Increased screening may reduce prevalence by increasing early treatment, but syphilis screening also has the potential to increase prevalence as individuals with latent syphilis are unlikely to transmit the infection to others unless they are treated and then infected again. Thus, we believe our estimates of infections averted and cost-effectiveness are conservative. Finally, there is limited data on population size, HIV and syphilis prevalence, and health seeking behaviours among key populations. We based our model input on estimates included in published literature as well as Viet Nam country sources.

## Conclusions

Our study suggests that annual or biannual HIV and syphilis testing among key populations in Viet Nam using a dual RDT will increase HIV and syphilis detection and treatment, while remaining cost saving or cost-effective. Integrating HIV and other STI testing can streamline services as well as expand testing and help countries with epidemics concentrated in key populations reach 95-95-95 targets. Future collection of empirical data, including conducting budget impact studies, would be useful to determine the impact of HIV and syphilis screening among key populations on ART uptake as well as HIV and syphilis incidence, particularly in concentrated HIV epidemics.

## Supplementary material

10.1136/bmjopen-2021-056887 online supplemental file 1

## Data Availability

Data are available on reasonable request.

## References

[1] UNAIDS Joint United Nations Programme on HIV/AIDS (2019). Global AIDS update 2019 - Communities at the centre. Geneva. https://www.unaids.org/en/resources/documents/2019/2019-global-AIDS-update.

[2] UNAIDS Joint United Nations Programme on HIV/AIDS (2020). Global AIDS update 2020: seizing the moment. Geneva. https://www.unaids.org/en/resources/documents/2020/global-aids-report.

[3] Cameron CE (2018). Syphilis vaccine development: requirements, challenges, and opportunities. Sex Transm Dis.

[4] Tsuboi M, Evans J, Davies EP (2021). Prevalence of syphilis among men who have sex with men: a global systematic review and meta-analysis from 2000-20. Lancet Glob Health.

[5] World Health Organization (2015). Consolidated guidelines on HIV testing services. Geneva. https://www.ncbi.nlm.nih.gov/books/NBK316021/.

[6] World Health Organization (2017). WHO guideline on syphilis screening and treatment for pregnant women. https://www.who.int/reproductivehealth/publications/rtis/syphilis-ANC-screenandtreat-guidelines/en/.

[7] Rodriguez PJ, Roberts DA, Meisner J (2021). Cost-Effectiveness of dual maternal HIV and syphilis testing strategies in high and low HIV prevalence countries: a modelling study. Lancet Glob Health.

[8] World Health Organization (2016). Consolidated guidelines on the use of antiretroviral drugs for treating and preventing HIV infection.

[9] UNAIDS (2016). 90-90-90 an ambitious treatment target to help end the AIDS epidemic. https://www.unaids.org/en/resources/909090.

[10] Hakim AJ, MacDonald V, Hladik W (2018). Gaps and opportunities: measuring the key population cascade through surveys and services to guide the HIV response. J Int AIDS Soc.

[11] World Health Organization (2016). Global health sector strategy on sexually transmitted infections 2016-2021. https://www.who.int/reproductivehealth/publications/rtis/ghss-stis/en/.

[12] World Health Organization Global health Observatory data Repository: data on syphilis. https://apps.who.int/gho/data/node.main.A1357STI?lang=en.

[13] Stover J, Brown T, Puckett R (2017). Updates to the Spectrum/Estimations and projections package model for estimating trends and current values for key HIV indicators. AIDS.

[14] Tuite AR, Testa C, Rönn M (2020). Exploring how epidemic context influences syphilis screening impact: a mathematical modeling study. Sex Transm Dis.

[15] Tuite A, Fisman D (2016). Go big or go home: impact of screening coverage on syphilis infection dynamics. Sex Transm Infect.

[16] Justumus P, Colby D, Mai Doan Anh T (2013). Willingness to use the Internet to seek information on HIV prevention and care among men who have sex with men in Ho Chi Minh City, Vietnam. PLoS One.

[17] Nguyen TA, Hoang LT, Pham VQ (2001). Risk factors for HIV-1 seropositivity in drug users under 30 years old in Haiphong, Vietnam. Addiction.

[18] Ngo AD, Ratliff EA, McCurdy SA (2007). Health-seeking behaviour for sexually transmitted infections and HIV testing among female sex workers in Vietnam. AIDS Care.

[19] World Health Organization (2019). Consolidated guidelines on HIV testing services. Geneva. https://www.who.int/publications/i/item/978-92-4-155058-1.

[20] Feldman J, Mishra S (2019). What could re-infection tell us about R_0_? A modeling case-study of syphilis transmission. Infect Dis Model.

[21] Edejer TT-T, Baltussen R, Adam T (2003). Making choices in health: who guide to cost effectiveness analysis.

[22] World Bank World Bank Data: GDP per capita (current US$) - Vietnam. https://data.worldbank.org/indicator/NY.GDP.PCAP.CD?locations=VN.

[23] Ong JJ, Fu H, Smith MK (2018). Expanding syphilis testing: a scoping review of syphilis testing interventions among key populations. Expert Rev Anti Infect Ther.

[24] UNAIDS Joint United Nations Programme on HIV/AIDS, WHO Laws and policies analytics.

[25] U.S. President’s Emergency Plan for AIDS Relief (PEPFAR) (2021). PEPFAR 2021 country and regional operational plan (COP/ROP) guidance for all PEPFAR countries. https://www.state.gov/wp-content/uploads/2020/12/PEPFAR-COP21-Guidance-Final.pdf.

[26] The Global Fund (2021). List of HIV diagnostic test kits and equipments classified according to the global fund quality assurance policy. https://www.theglobalfund.org/media/5878/psm_productshiv-who_list_en.pdf.

[27] Broyles LN, Boeras D, Peeling RW (2021). Implementation of dual maternal HIV-Syphilis testing: the devil is in the details. Lancet Glob Health.

[28] Nguyen V, Anh L, Thong N (2019). Poster exhibition IAS 2019.

[29] Granich RM, Gilks CF, Dye C (2009). Universal voluntary HIV testing with immediate antiretroviral therapy as a strategy for elimination of HIV transmission: a mathematical model. Lancet.

[30] Eaton JW, Johnson LF, Salomon JA Hiv treatment as prevention: systematic comparison of mathematical models of the potential impact of antiretroviral therapy on HIV incidence in South Africa. PLoS Med.

[31] UNAIDS AIDSinfo | UNAIDS. https://aidsinfo.unaids.org/.

[32] Iwuji CC, Orne-Gliemann J, Larmarange J (2018). Universal test and treat and the HIV epidemic in rural South Africa: a phase 4, open-label, community cluster randomised trial. Lancet HIV.

[33] Makhema J, Wirth KE, Pretorius Holme M (2019). Universal testing, expanded treatment, and incidence of HIV infection in Botswana. N Engl J Med.

[34] Havlir DV, Balzer LB, Charlebois ED (2019). Hiv testing and treatment with the use of a community health approach in rural Africa. N Engl J Med.

[35] Hayes RJ, Donnell D, Floyd S (2019). Effect of Universal Testing and Treatment on HIV Incidence - HPTN 071 (PopART). N Engl J Med.

[36] Akullian A, Bershteyn A, Jewell B (2017). The missing 27. AIDS.

[37] Abdool Karim SS (2019). HIV-1 Epidemic Control - Insights from Test-and-Treat Trials. N Engl J Med.

[38] Swartzendruber A, Steiner RJ, Adler MR (2015). Introduction of rapid syphilis testing in antenatal care: a systematic review of the impact on HIV and syphilis testing uptake and coverage. Int J Gynaecol Obstet.

[39] Gliddon HD, Peeling RW, Kamb ML (2017). A systematic review and meta-analysis of studies evaluating the performance and operational characteristics of dual point-of-care tests for HIV and syphilis. Sex Transm Infect.

[40] Kato M, Long NH, Duong BD (2014). Enhancing the benefits of antiretroviral therapy in Vietnam: towards ending AIDS. Curr HIV/AIDS Rep.

[41] Kato M, Granich R, Bui DD (2013). The potential impact of expanding antiretroviral therapy and combination prevention in Vietnam: towards elimination of HIV transmission. J Acquir Immune Defic Syndr.

[42] Kazemian P, Costantini S, Kumarasamy N (2020). The cost-effectiveness of human immunodeficiency virus (HIV) preexposure prophylaxis and HIV testing strategies in high-risk groups in India. Clin Infect Dis.

[43] Cipriano LE, Zaric GS, Holodniy M (2012). Cost effectiveness of screening strategies for early identification of HIV and HCV infection in injection drug users. PLoS One.

[44] Reitsema M, Steffers L, Visser M (2019). Cost-Effectiveness of increased HIV testing among MSM in the Netherlands. AIDS.

[45] Green KE, Vu BN, Phan HT (2018). From conventional to disruptive: upturning the HIV testing status quo among men who have sex with men in Vietnam. J Int AIDS Soc.

[46] Withers K, Bristow C, Nguyen M (2019). A field evaluation of a rapid dual immunoassay for human immunodeficiency virus and syphilis antibodies, Hanoi, Vietnam. Int J STD AIDS.

[47] Vu BN, Green KE, Thi Thu Phan H (2018). Lay provider HIV testing: a promising strategy to reach the undiagnosed key populations in Vietnam. PLoS One.

[48] Nguyen VTT, Phan HT, Kato M (2019). Community-led HIV testing services including HIV self-testing and assisted partner notification services in Vietnam: lessons from a pilot study in a concentrated epidemic setting. J Int AIDS Soc.

[49] Clatts MC, Goldsamt LA, Giang LM (2016). Sexually transmissible infection and HIV prevention and treatment for young male sex workers in Vietnam: findings from the sheath intervention. Sex Health.

[50] Bao A, Colby DJ, Trang T (2016). Correlates of HIV testing among transgender women in Ho Chi Minh, Vietnam. AIDS Behav.

[51] Magnani R, Riono P, Saputro E (2010). Sexual risk behaviours, HIV and other sexually transmitted infections among female sex workers in Indonesia. Sex Transm Infect.

[52] Cassini A, Colzani E, Pini A (2018). Impact of infectious diseases on population health using incidence-based disability-adjusted life years (DALYs): results from the burden of communicable diseases in Europe study, European Union and European economic area countries, 2009 to 2013. Euro Surveill.

