# Supplementary material for: Cost-effectiveness of implementing HIV and HIV/syphilis dual testing among key populations in Viet Nam: a modelling analysis
Source: BMJ Open. 2022 Aug 11;12(8):e056887. doi: 10.1136/bmjopen-2021-056887 (PMC9379490; doi:10.1136/bmjopen-2021-056887)
Supplement: Supplementary file 2 [file bmjopen-2021-056887.reviewer_comments.pdf]

PEER REVIEW HISTORY

BMJ Open publishes all reviews undertaken for accepted manuscripts. Reviewers are asked to complete a checklist review form (**http://bmjopen.bmj.com/site/about/resources/checklist.pdf**) and are provided with free text boxes to elaborate on their assessment. These free text comments are reproduced below.

ARTICLE DETAILS

**TITLE (PROVISIONAL)**	Cost-effectiveness of implementing HIV and HIV/syphilis dual testing among key populations in Viet Nam: a modeling analysis
**AUTHORS**	Coomes, David; Green, Dylan; Barnabas, Ruanne; Sharma, Monisha; Barr-DiChiara, Magdalena; Jamil, Muhammad; Baggaley, R; Owiredu, Morkor Newman; Macdonald, Virginia; Nguyen, Van Thi Thuy; Vo, Son; Taylor, Melanie; Wi, Teodora; Johnson, Cheryl; Drake, Alison L.

VERSION 1 – REVIEW

**REVIEWER**	Bilal, .<br>Hubei University of Economics
**REVIEW RETURNED**	04-Jan-2022

**GENERAL COMMENTS**	Thank you very much for the opportunity to review this manuscript. The manuscript addressed a significant issue, and the article is well constructed. However, I have the following comments to improve this paper:<br>1) The introduction is concise. I urge the authors to expand it.<br>2) The authors highlight this paper's incremental contributions to the literature.<br>3) I recommend adding the conclusions section. The authors must include future research implications in detail<br>4) Finally, the authors check the minor issues and typos.

**REVIEWER**	Cassels, S<br>University of California Santa Barbara
**REVIEW RETURNED**	28-Jan-2022

**GENERAL COMMENTS**	Thank you for the opportunity to review the BMJ submission “Cost-effectiveness of implementing HIV and HIV/syphilis dual testing among key populations in Viet Nam: a modeling analysis.” Overall, I found this analysis to be very important and timely, with robust findings. The strengths of the paper include 1) a clear motivation, literature, and background for the modeling exercise and scenario selection, and 2) strong and robust costing and cost effectiveness analysis. I have slight concerns about the model parametrization and the way testing is conceptualized.<br><br>Overall, I have a high confidence in this paper and the results. The authors conclude that most cost savings comes from averting HIV than syphilis, but it is still best to use the dual test. The rigorous sensitivity analyses, especially, provide confidence in the results. The costing data is robust and clear, as are most of the parameters point estimates and assumptions.

	My main concern has to do with the model conceptualization of changes in testing rates and test uptake. The paper states ”We modeled increases in testing coverage by adjusting the percent of adults living with HIV (PLWH) on ART.” I was slightly surprised that testing was not an explicitly modeled process; rather, changes in testing levels and uptake were examined by assuming different levels or ART coverage. I assume the baseline SPECTRUM model structure does not allow for changes in testing (is this correct?) There are a lot of steps and assumptions between changes in testing behavior and ART coverage, but the authors do not really address this as a limitation.<br><br>One small comment: as someone who is not deeply familiar with the Spectrum AIDS impact model, I would have liked a quick overview on the important model components, assumptions, and limitations. A paragraph dedicated to the model might be useful: pros/cons on using a deterministic compartmental model for the entirety of Viet Nam, do different at risk groups interact?, what are the key drivers of transmission, are there any spatial components?<br><br>For example, how is the timing of annual or biannual testing implemented in the model? Since this is a deterministic model, there are a few ways this could be implemented: is there a change in the rate of ART uptake? Or is there a “jump” or break in the level of ART coverage once or twice a year? I can imagine that these two implementation strategies could lead to different conclusions.<br><br>A figure of incidence over time might be useful, with indicators of when testing scenarios are implemented. This might help clarify how the timing of the interventions works, and to see the nature of the impact in reduced incidence. For example, are the cases averted right after lots of people are tested (a blip?) or are they averted gradually over time (a continuous impact?).

**REVIEWER**	Sousa, João<br>KU Leuven, Department of Microbiology and Immunology
**REVIEW RETURNED**	07-Feb-2022

**GENERAL COMMENTS**	p.8. In the Introduction, or elsewhere, you should describe technical aspects of the tests that are being implemented. For example, in the case of syphilis, there are two broad categories of tests: non-treponemal (RPR, VDRL, etc) or treponemal (TPHA, FTA-ABS, etc). Normally, the latter are confirmatory. The gold standard is a non-treponemal followed by a confirmatory treponemal, and if they are both positive, that indicates active syphilis. If you are implementing only RPR alone, you should justify that choice. Similarly for HIV, you could say more about the kind of test, Elisa, confirmatory Western Blot, or other.<br>p.9. In Methods, it seems more appropriate to refer to the package used (Spectrum) and other general methods used first, and only after to present Table 1 with the parameters.<br>p.11. Here you mention that RPR is the “initial syphilis test”; this suggests that a treponemal confirmatory test is done after; please explain if that is the case, somewhere in the article.<br>p.11. You mention penicillin treatment; however, often the treatment is with Benzathine penicillin G; if so, please mention it.<br>p.12. You assume that syphilis epidemic is not affected by testing and treatment. However, it is likely to be affected. If people treat themselves early on first symptoms, the latter will be cleared, and

	further transmission avoided. Please justify why you make this assumption.<br><br>Minor corrections<br>p.4. Please define "DALY" on your first use in the Abstract<br>p.8. You use "FSW" for the first time, of course it is female sex workers, but you should write that. Previously you used only "SW" for sex workers.<br>p.8. Better "higher than *in* the general population"<br>p.9. Please define "DALY" on your first use out of the Abstract<br>p.16. Please define "PEPFAR" on your first use

VERSION 1 – AUTHOR RESPONSE

Reviewer #1:

REVIEWER 1, OVERALL COMMENTS: Thank you very much for the opportunity to review this manuscript. The manuscript addressed a significant issue, and the article is well constructed.
We appreciate the reviewer's positive comments on our manuscript.

REVIEWER 1, COMMENT 1: The introduction is concise. I urge the authors to expand it.

We appreciate the reviewer's comment and have slightly expanded the introduction. While we would have liked to include an even more comprehensive introduction, due to word limitations for readability we have chosen a more concise introduction to allow for a longer discussion.

REVIEWER 1, COMMENT 2: The authors highlight this paper's incremental contributions to the literature.

We thank the reviewer for their comment and acknowledgement of the contributions to the literature.

REVIEWER 1, COMMENT 3: I recommend adding the conclusions section. The authors must include future research implications in detail.
We appreciate the reviewer's helpful comment and have added additional language in the conclusions section that outlines specific future research needs.

REVIEWER 1, COMMENT 4: Finally, the authors check the minor issues and typos.
We thank the reviewer for pointing this out. We have further edited the manuscript for these issues.

Reviewer #2:
REVIEWER 2, OVERALL COMMENTS: Thank you for the opportunity to review the BMJ submission "Cost-effectiveness of implementing HIV and HIV/syphilis dual testing among key populations in Viet Nam: a modeling analysis." Overall, I found this analysis to be very important and timely, with robust findings. The strengths of the paper include 1) a clear motivation, literature, and background for the modeling exercise and scenario selection, and 2) strong and robust costing and cost effectiveness analysis. I have slight concerns about the model parametrization and the way testing is conceptualized.

Overall, I have a high confidence in this paper and the results. The authors conclude that most cost savings comes from averting HIV than syphilis, but it is still best to use the dual test. The rigorous

sensitivity analyses, especially, provide confidence in the results. The costing data is robust and clear, as are most of the parameters point estimates and assumptions.
We appreciate the reviewer's positive comments on our manuscript.

REVIEWER 2, COMMENT 1: My main concern has to do with the model conceptualization of changes in testing rates and test uptake. The paper states "We modeled increases in testing coverage by adjusting the percent of adults living with HIV (PLWH) on ART." I was slightly surprised that testing was not an explicitly modeled process; rather, changes in testing levels and uptake were examined by assuming different levels or ART coverage. I assume the baseline SPECTRUM model structure does not allow for changes in testing (is this correct?) There are a lot of steps and assumptions between changes in testing behavior and ART coverage, but the authors do not really address this as a limitation.

We agree with the reviewer's comment that this is a limitation of the model and have added additional detail in the limitations section of the manuscript, starting on line 350. The reviewer is correct in assuming that SPECTRUM does not allow for changes in testing rates, and that is why we chose to model ART coverage rates as a proxy.

REVIEWER 2, COMMENT 2: One small comment: as someone who is not deeply familiar with the Spectrum AIDS impact model, I would have liked a quick overview on the important model components, assumptions, and limitations. A paragraph dedicated to the model might be useful: pros/cons on using a deterministic compartmental model for the entirety of Viet Nam, do different at risk groups interact?, what are the key drivers of transmission, are there any spatial components?

We have updated the methods section with a short overview on SPECTRUM (in the first paragraph of methods), to include model components. This model does include interactions between groups, and we describe this briefly in the methods addition.
To discuss pros/cons of using this model and spatial components we have included additional explanation in the limitations section:
"Research that focuses on province-specific estimates of cost and impact would likely find that focusing on high-burden areas is more cost-effective, however, health policy, financing, guideline development, and implementation continue to be led nationally in Viet Nam therefore national-level evidence is needed to direct decision making."
While we agree with the reviewer that a province-specific model may better describe the epidemic in Viet Nam and provide better cost-effectiveness estimates, we balance this with several other factors. First, province-specific estimates of important parameters (e.g., prevalence) are not available for key populations in all 63 provinces. Our model therefore represents a sort of "average" impact and cost-effectiveness. We feel this is a balance between data availability and compromise of national-level estimation. Second, control over province level vs. national level interventions is a sensitive topic in Viet Nam and, based on feedback from our in-country partners and authors, we chose to analyze our interventions at the natioal level.

REVIEWER 2, COMMENT 3: For example, how is the timing of annual or biannual testing implemented in the model? Since this is a deterministic model, there are a few ways this could be implemented: is there a change in the rate of ART uptake? Or is there a "jump" or break in the level of ART coverage once or twice a year? I can imagine that these two implementation strategies could lead to different conclusions.
There is a break (or jump) in ART coverage from year-to-year, as SPECTRUM inputs are yearly ART coverage, with higher ART changes for the two HIV test scenarios as compared to the one HIV test scenarios. But these changes are smoothed over time – we assume that ART coverage increases at a constant rate per year until it reaches 95% (with higher rates for more testing). Based on

your comment, we add more discussion to the limitations of this assumption in the paragraph starting on line 360.

REVIEWER 2, COMMENT 4: A figure of incidence over time might be useful, with indicators of when testing scenarios are implemented. This might help clarify how the timing of the interventions works, and to see the nature of the impact in reduced incidence. For example, are the cases averted right after lots of people are tested (a blip?) or are they averted gradually over time (a continuous impact?).

We thank the reviewer for this helpful feedback and have included a figure of HIV incidence over time in the appendix (Figure S1). Based on model inputs, the intervention is not carried out at a single time point per year (i.e. everyone is not tested on January 1). The increased ART uptake that we use to model testing results in a higher probability that an individual in the model will initiate ART throughout the year, resulting in (fairly) smooth changes in incidence. Inputs and outputs for the model are all reported in yearly intervals, resulting in cases averted gradually over time.

Reviewer #3

REVIEWER 3, COMMENT 1: p.8. In the Introduction, or elsewhere, you should describe technical aspects of the tests that are being implemented. For example, in the case of syphilis, there are two broad categories of tests: non-treponemal (RPR, VDRL, etc) or treponemal (TPHA, FTA-ABS, etc). Normally, the latter are confirmatory. The gold standard is a non-treponemal followed by a confirmatory treponemal, and if they are both positive, that indicates active syphilis. If you are implementing only RPR alone, you should justify that choice. Similarly for HIV, you could say more about the kind of test, Elisa, confirmatory Western Blot, or other.
We thank the reviewer for this comment, and we agree that a TPHA confirmatory syphilis test should be included in the model. We have updated the model to include this test and describe this addition in the methods section. Additionally, based on current practices in Viet Nam, all members of key populations who test positive using a dual HIV/syphilis test are also tested using an RPR and TPHA test (which is usually conducted at a regional hospital). We have also specified that we include HIV RDT tests in the model and the rationale, as follows:
"We modeled HIV RDTs as WHO recommend, limiting the use of lab-based testing such as western blot, especially in hard-to-reach populations, to increase access and limit the loss to follow up."

REVIEWER 3, COMMENT 2: p.9. In Methods, it seems more appropriate to refer to the package used (Spectrum) and other general methods used first, and only after to present Table 1 with the parameters.
We agree with the reviewer and have updated the methods section accordingly.

REVIEWER 3, COMMENT 3: p.11. Here you mention that RPR is the "initial syphilis test"; this suggests that a treponemal confirmatory test is done after; please explain if that is the case, somewhere in the article.

We have updated the model to include a TPHA confirmatory syphilis test (see comment 1 for more detail).

REVIEWER 3, COMMENT 4: p.11. You mention penicillin treatment; however, often the treatment is with Benzathine penicillin G; if so, please mention it.

We agree with the reviewer and have updated the manuscript to specify Benzathine penicillin G.

REVIEWER 3, COMMENT 5: p.12. You assume that syphilis epidemic is not affected by testing and treatment. However, it is likely to be affected. If people treat themselves early on first symptoms, the latter will be cleared, and further transmission avoided. Please justify why you make this assumption.

We agree with the reviewer that it is possible early treatment of syphilis can result in the avoidance of further transmission. However, the impact of syphilis screening on prevalence within populations is not clear. A few mathematical modeling studies have recently estimated that syphilis prevalence may increase, decrease, or stay constant depending on screening rates and community characteristics. We have added the following language to the methods section starting on line 129 (with references):
"This model assumes that syphilis testing and treatment does not impact syphilis prevalence, although increased screening could potentially result in reduced, unchanged, or increased syphilis prevalence depending on coverage."
These prior studies have postulated that additional screening could result in cases of latent syphilis being treated, thereby increasing the number of susceptible individuals in the population. These susceptible individuals may then be reinfected and pose the potential to pass along active syphilis to another.
Based on this reasoning, reduced syphilis prevalence (if it occurs) would be an unaccounted-for benefit from these modeled interventions. Having said that, reduced syphilis prevalence may make the interventions less cost-effective, however, as most of the benefits (>98% of DALYs) are realized from HIV testing, they are unlikely to largely impact this. Additionally, evidence has shown that if syphilis prevalence is reduced due to increased screening, it quickly bounces back once screening is lowered, possibly making syphilis screening under low syphilis prevalence desirable.
Due to the uncertainty in the literature on the impact of case finding and prevalence, we choose to keep the syphilis prevalence stable in our model population as empirical information on the impact of syphilis screening on population prevalence is not available. Our manuscript has been updated with these sources (from the added line above).

REVIEWER 3, COMMENT 6:
Minor corrections
p.4. Please define “DALY” on your first use in the Abstract
p.8. You use “FSW” for the first time, of course it is female sex workers, but you should write that. Previously you used only “SW” for sex workers.
p.8. Better “higher than *in* the general population”
p.9. Please define “DALY” on your first use out of the Abstract
p.16. Please define “PEPFAR” on your first use
Thank you for pointing these out – they have all been corrected in the manuscript as suggested.

VERSION 2 – REVIEW

**REVIEWER**	Cassels, S<br>University of California Santa Barbara
**REVIEW RETURNED**	23-Jun-2022

**GENERAL COMMENTS**	The authors have thoroughly and thoughtfully responded to my comments and concerns.
